# Colon tissue-accumulating mesoporous carbon nanoparticles loaded with *Musca domestica* cecropin for ulcerative colitis therapy

**DOI:** 10.7150/thno.53105

**Published:** 2021-01-19

**Authors:** Lun Zhang, Shuiqing Gui, Yinghua Xu, Jiali Zeng, Jian Wang, Qingru Chen, Liqian Su, Ziyan Wang, Rui Deng, Fujiang Chu, Wenbin Liu, Xiaobao Jin, Xuemei Lu

**Affiliations:** 1 Guangdong Provincial Key Laboratory of Pharmaceutical Bioactive Substances, School of Life Science and Biopharmaceutics, Guangdong Pharmaceutical University, 280 Wai Huan Dong Road, Guangzhou Higher Education Mega Center, Guangzhou 510006, People's Republic of China.; 2 Intensive Care Unit, Shenzhen Second People's Hospital, the First Affiliated Hospital of Shenzhen University, Shenzhen 518031, People's Republic of China.; 3 Key Laboratory of the Ministry of Health for Research on Quality and Standardization of Biotech Products, National Institutes for Food and Drug Control, Beijing 102629, People's Republic of China.; 4 School of Pharmacy, Guangdong Pharmaceutical University, 280 Wai Huan Dong Road, Guangzhou Higher Education Mega Center, Guangzhou 510006, People's Republic of China.

**Keywords:** ulcerative colitis, *Musca domestica* cecropin, mesoporous carbon nanoparticles, colon-accumulating drug delivery, enhanced therapy

## Abstract

Ulcerative colitis (UC) is a modern refractory disease with steadily increasing incidence worldwide that urgently requires effective and safe therapies. Therapeutic peptides delivered using nanocarriers have shown promising developments for the treatment of UC. We developed a novel colon-accumulating oral drug delivery nanoplatform consisting of *Musca domestica* cecropin (MDC) and mesoporous carbon nanoparticles (MCNs) and investigated its effects and mechanism of action for the treatment of UC.

**Methods:** An optimized one-step soft templating method was developed to synthesize MCNs, into which MDC was loaded to fabricate MDC@MCNs. MCNs and MDC@MCNs were characterized by BET, XRD, and TEM. MDC and MDC@MCNs resistance to trypsin degradation was measured through Oxford cup antibacterial experiments using *Salmonella typhimurium* as the indicator. Uptake of MDC and MDC@MCNs by NCM460 cells was observed by fluorescence microscopy. The biocompatibility of MDC, MCNs, and MDC@MCNs was evaluated in three cell lines (NCM460, L02, and NIH3T3) and C57BL/6 mice. Dextran sulphate sodium was used to establish models of NCM460 cell injury and UC in mice. MTT assay, flow cytometry, and mitochondrial membrane potential assay were applied to determine the effects of MDC@MCNs on NCM460 cells injury. Additionally, a variety of biological methods such as H&E staining, TEM, ELISA, qPCR, Western blotting, and 16s rDNA sequencing were performed to explore the effects and underlying mechanism of MDC@MCN on UC *in vivo*. Colonic adhesion of MCNs was compared in normal and UC mice. The oral biodistributions of MDC and MDC@MCNs in the gastrointestinal tract of mice were also determined.

**Results:** MDC@MCNs were successfully developed and exhibited excellent ability to resist destruction by trypsin and were taken up by NCM460 cells more readily than MDC. *In vitro* studies showed that MDC@MCNs better inhibited DSS-induced NCM460 cells damage with lower toxicity to L02 and NIH3T3 cells compared with MDC. *In vivo* results indicated that MDC@MCNs have good biocompatibility and significantly improved colonic injury in UC mice by effectively inhibiting inflammation and oxidative stress, maintaining colonic tight junctions, and regulating intestinal flora. Moreover, MDC@MCNs were strongly retained in the intestines, which was attributed to intestinal adhesion and aggregation of MCNs, serving as one of the important reasons for its enhanced efficacy after oral administration compared with MDC.

**Conclusion:** MDC@MCNs alleviated DSS-induced UC by ameliorating colonic epithelial cells damage, inhibiting inflammation and oxidative stress, enhancing colonic tight junctions, and regulating intestinal flora. This colon-accumulating oral drug delivery nanoplatform may provide a novel and precise therapeutic strategy for UC.

## Introduction

Ulcerative colitis (UC) is a chronic and recurrent inflammatory bowel disease (IBD) that is listed as one of the modern refractory diseases by the World Health Organization (WHO) 1. UC is characterized with non-specific inflammatory response and ulcer formation in colorectal mucosa, which causes various complications such as stomach pain, diarrhea and rectal bleeding 2. Patients with UC also have an increased risk of developing colorectal cancer as a result of long-term exposure to the chronic inflammatory environment and repeated cycles of damage and repair 3. Although many efforts have been expended to clarify the possible pathogenesis of UC, the precise causes for its incidence remain to be uncovered 4. At present, hormones, anti-inflammatory chemical drugs, and immunosuppressive agents effectively inhibit inflammation in UC; however, their utility has been greatly limited by unsatisfactory long-term efficacy, drug resistance, and severe systemic side effects 5. Therefore, there is an urgent need for novel effective and sustained treatments with minimal side effects for UC.

In the last decade, peptides have increasingly been considered a main treatment strategy along with small molecules and biologics. A wide variety of new peptide medicines have been developed on a commercial scale as a result of advances in the biotechnology field 6. In the treatment of UC, peptide drugs have shown promising developments 7. In a previous study, we demonstrated that *Musca domestica* cecropin (MDC), a novel 40 amino acid antimicrobial peptide (AMP), possesses potential antibacterial, anti-inflammatory, and immunological functions and potent protection against colonic mucosal barrier impairment induced by Salmonella typhimurium in mice 8. Although the exact pathogenesis of UC remains unclear, it is worth noting that mounting studies have demonstrated that immune disorders, genetic susceptibility, flora disturbances, and many other factors are involved 9-14. Hence, it is of great significance and possible to explore the role and mechanism of MDC in the treatment of UC. However, MDC has several inherent limitations to UC treatment including poor oral bioavailability and instability 15. Developing MDC into an oral preparation is important because oral administration provides convenience, high patient compliance, and cost-effectiveness 16. In addition, the main aim of UC therapy is to control inflammation, achieve mucosal healing, maintain remission, and reduce surgeries and hospitalizations 17. Thus, the inability of MDC to accumulate at the ulcer site is another challenge for its effective treatment of UC.

Nanomedicine has made great progress in the treatment of colitis and tumors 18-22, providing the direction for the development of peptide drugs as nano preparations. To address problems in the oral delivery of peptide drugs, various nanocarriers have been explored including lipid nanoparticles, microemulsions, micelles and polymeric nanoparticles 23-25. An oral colon-specific drug delivery system consisting of an appropriate nanocarrier and peptide drug is of great interest for UC therapy as it can provide multiple advantages over free peptide including increased bioavailability, lower toxicity, and higher therapeutic efficacy. Suitable nanocarriers for MDC should be assessed for high peptide loading, stability in the gastrointestinal tract, and selective retention in colitis tissue. In recent years, carbon nanomaterials such as nanotubes, nanofibers, nanospheres, and fullerenes have attracted growing attention for energy, interfacial catalysis, and drug delivery applications 26-31. In particular, mesoporous carbon nanoparticles (MCNs) with their large pore volumes and high specific surface areas possess several advantageous characteristics for drug delivery including controllable drug release, modifiable targeting, and high drug loading ratios. Lin et al. fabricated MCNs as a potential oral delivery system of insulin to reduce adverse reactions from hypodermic injection 32. Furthermore, MCNs have been confirmed to enhance the oral bioavailability of amphiphobic drugs via lymphatic transport tropism and ileum bioadhesion 33. Hence, it is reasonable to design a safe colon-accumulating oral delivery system for MDC based on the potential intestinal adhesion of MCNs.

However, it remains unclear whether MDC can benefit the treatment of UC and the underlying mechanism is unknown. Moreover, whether MCNs can safely enhance the delivery and therapeutic efficacy of MDC requires investigation. Thus, this study aims to synthesize MCNs using a simple, efficient, low-cost and pollution-free method; to design an effective colon-accumulating oral delivery system for MDC based on MCNs (MDC@MCNs); and to evaluate the safety, therapeutic efficacy on UC, and underlying mechanism of MDC@MCNs *in vitro* and *in vivo.*

## Results and Discussion

### Preparation and characterization of MCNs and MDC@MCNs

**Figure 1:**
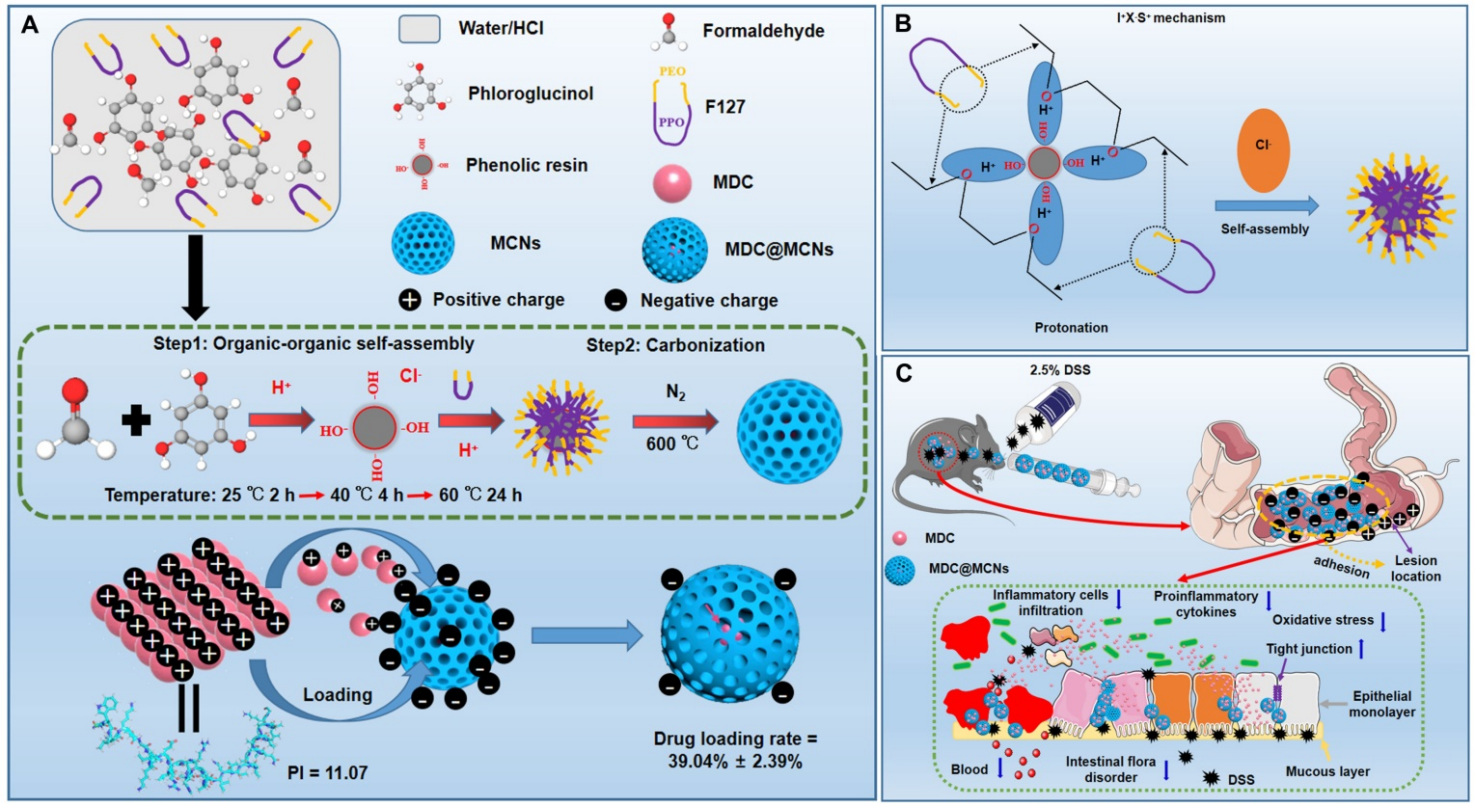
** (A)** Synthesis procedure and schematic illustration of shape evolution path of MCNs as well as construction of MDC-loaded nano-platform based on MCNs (MDC@MCNs). **(B)** I^+^X^-^S^+^ mechanism of MCNs synthesis by self-assembly. **(C)** Schematic diagram of MDC@MCNs accumulating and releasing drugs in the diseased colon for enhanced UC therapy.

Due to the merits of low density, high porosity, and large specific surface area, mesoporous carbon has displayed great promising in various areas especially drug delivery 34-39. However, most reported mesoporous carbons characterized with large size, irregular shape and small pore size, which largely hinder their further applications 40, 41. The development of a simple and efficient synthesis method producing MCNs that are suitable as drug carriers is a long-pursued objective. The methods used to synthesize MCNs are commonly divided into hard-template method and organic-organic self-assembly soft-template methods 42. The latter methods involve faster and simpler procedures and produce products with adjustable particle size and clear macroscopic morphology 43. Nevertheless, these strategies are usually limited by the narrow pH range in the reaction system and require an additional pre-polymerization step for obtaining low molecular weight phenolic resoles 44, 45. Hydrogen and coulombic interactions between phenolic resin precursors and amphiphilic block copolymers can be facilitated and enhanced by acidity, making acidic conditions preferred over alkaline conditions in the preparation of MCNs 46. However, rapid polymerization of the carbon source under strongly acidic conditions creates difficulties in the direct soft-template synthesis by self-assembly of a copolymer and carbon precursors 47. Therefore, MCNs are rarely prepared under acidic conditions through direct self-assembly methods. In the present work, we demonstrate a facile, effective, and optimized one-step soft templating method under acidic conditions to fabricate MCNs with a small particle size and large pore size. We capitalized on triblock copolymer F127 as the template for mesopore formation and aid in dispersal and stability, and phloroglucinol/formaldehyde (PF) as a carbon precursor without any auxiliary additives (**Figure 1A**). As shown in **Figure 1B**, ethylene oxide (EO) blocks of F127 and PF were protonated under acidic conditions. Coulombic interaction in the self-assembly of F127-PF was induced through the I^+^X^-^S^+^ mechanism with Cl^-^ acting as the mediator 46. F127 decomposed to generate mesopores, and PF was converted to the carbon framework during carbonization.

**Figure 2:**
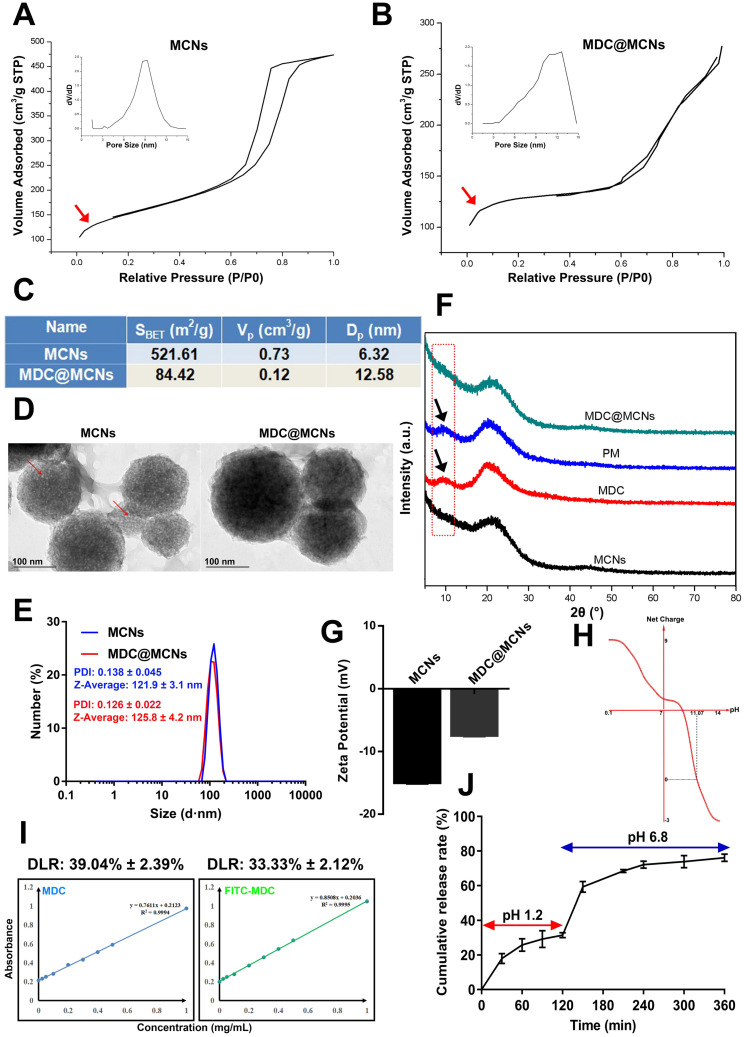
Characterization of MCNs and MDC@MCNs. **(A)-(B)** The nitrogen adsorption/desorption isotherm and its corresponding PSD curve of MCNs and MDC@MCNs. **(C)** Physical property parameters of MCNs and MDC@MCNs obtained from BET test including specific surface area (S_BET_), pore volume (Vp) and pore size distribution (Dp). **(D)** Representative TEM image of MCNs and MDC@MCNs, and arrows point to the hole of MCNs. **(E)** Particle size of MCNs and MDC@MCNs. **(F)** The XRD pattern of MCNs, MDC, PM and MDC@MCNs. **(G)** Zeta potential of MCNs and MDC@MCNs, values are means ± SEMs, n = 3. **(H)** The change of the net charge of MDC with pH variation. **(I)** MDC and FITC-MDC content detection standard curves. The drug loading rates (DLR) of MDC@MCNs and FITC-MDC@MCNs are 39.04% ± 2.39% and 33.33% ± 2.12% respectively, values are means ± SEMs, n = 3. **(J)** The continuous release of MDC from MDC@MCNs in solution (pH 1.2 and 6.8) for 6 h, values are means ± SEMs, n = 3.

MDC@MCNs were prepared by the principle of physical adsorption and charge interaction. The N_2_-sorption isotherms and pore-size distributions (PSD) of MCNs and MDC@MCNs are shown in **Figure 2A-B**. The type IV N_2_-sorption isotherms of MCNs indicate that they are mesoporous materials. The capillary condensation stage of the N_2_-sorption isotherms of MDC@MCNs is significantly slower than that of MCNs without MDC loading and there is no hysteresis loop, indicating that the mesoporous characteristics of the samples disappeared. Moreover, **Figure 2A-C** reveal that the PSD of MDC@MCNs is wider and shifts to the right compared with that of MCNs, and the specific surface area (521.61 *vs* 84.42 m^2^/g) and pore volume (0.73 *vs* 0.12 cm^3^/g) decreased significantly, indicating that MDC was loaded into the MCNs channels. To further determine the morphology and particle size of MCNs and whether MDC was fully loaded, TEM and XRD were carried out. The corresponding results (**Figure 2D-E**) illustrate that MCNs are ~120 nm in size, spherical in shape, and have many mesopores on their surfaces. The obvious visual difference between MCNs and MDC@MCNs is that the holes on the surface of MDC@MCNs are filled. MCNs and MDC@MCNs have approximate average diameters and polydispersity indexes (PDI). The XRD results (**Figure 2F**) clearly show that MDC and a physical mixture (PM) possess an obvious characteristic diffraction peak at 10° (2θ), while MCNs and MDC@MCNs do not. Therefore, the XRD experiment validated the previous characterization results, implying that MCNs and MDC@MCNs were synthesized successfully.

Additionally, we established standard curves for MDC and FITC-MDC detection. By using the standard curves (**Figure 2I**), the drug loading rates of MDC@MCNs and FITC-MDC@MCNs were determined to be 39.04% ± 2.39% and 33.33% ± 2.12%, respectively. These reasonably high drug loading rates may be attributed to a certain interaction between the positive charge of MDC (**Figure 2H**) and the negative charge of MCNs (**Figure 2G**) in double distilled water. MDC release from MCNs was measured in artificial gastric fluid (pH 1.2) and intestinal fluid (pH 6.8), which simulated the continuous release process *in vivo* after oral administration. The cumulative release curve (**Figure 2J**) shows that 76.21% of MDC was released in 6 h, of which 31.17% was released in the first 2 h in artificial gastric fluid and 45.04% was released in the last 4 h in artificial intestinal fluid. With the change in release medium and corresponding pH increase from 1.2 to 6.8, drug release slightly accelerated. This acceleration is attributed to the decrease in positive net charge of MDC (**Figure 2H**) and weakening of the MDC-MCNs charge interaction.

### MDC@MCNs resist activity damage induced by trypsin and possess more entry into NCM460 cells than MDC

**Figure 3:**
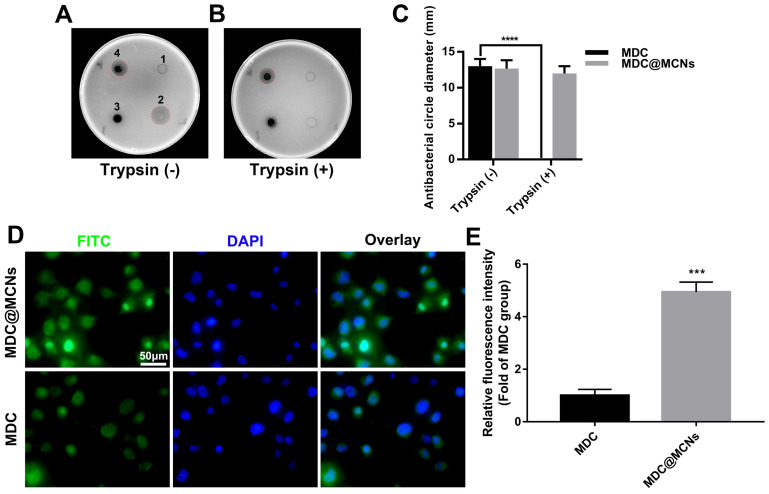
MDC@MCNs resist activity damage induced by trypsin and possess more entry into NCM460 cells than MDC.** (A)-(B)** Representative bacteriostasis plates of normal saline (1), MDC (2), MCNs (3) and MDC@MCNs (4) against *Salmonella typhimurium.* Trypsin (+) and Trypsin (-) represent that the experimental drugs above are treated with and without trypsin respectively. **(C)** Statistical diagram of bacteriostatic ring size of MDC and MDC@MCNs before and after trypsin treatment, values are means ± SEMs, n = 3. ****p < 0.0001. **(D)** Representative fluorescence localization pictures of FITC-MDC and FITC-MDC@MCNs uptake by NCM460 cells. **(E)** Relative fluorescence intensity statistical graph reflecting the cellular uptake, values are means ± SEMs, n = 3. ***p < 0.001.

MDC can inhibit *Salmonella typhimurium*. Thus, the size of bacteriostatic circle was used to estimate the MDC activity. As shown in **Figure 3A**, there was no bacteriostatic circle when normal saline (**1**) was added as negative control. MDC (**2**) without trypsin treatment displayed good antibacterial activity and produced a 13 mm bacteriostatic circle. As expected, MCNs (**3**) had no activity against *Salmonella typhimurium*. It was gratifying that MDC@MCNs (**4**) exhibited the same activity as MDC, which indirectly demonstrated that MDC could be completely released from MCNs under these experimental conditions. However, after trypsin treatment, it was a different case that MDC activity was destroyed completely (**Figure 3B-C**). It was surprising that the activity of MDC@MCNs was retained with trypsin treatment, which may be attributed to protection of MDC within MCNs pores from trypsin-induced damage. In addition, compared with MDC, MDC@MCNs were more easily ingested by NCM460 normal human colon mucosal epithelial cells, resulting in release of the same amount of MDC (**Figure 3D-E**). From this result, we expect MCNs to be an effective carrier of MDC in the treatment of NCM460 cells damage.

### Cytotoxicity to NCM460, L02, and NIH3T3 cells

**Figure 4:**
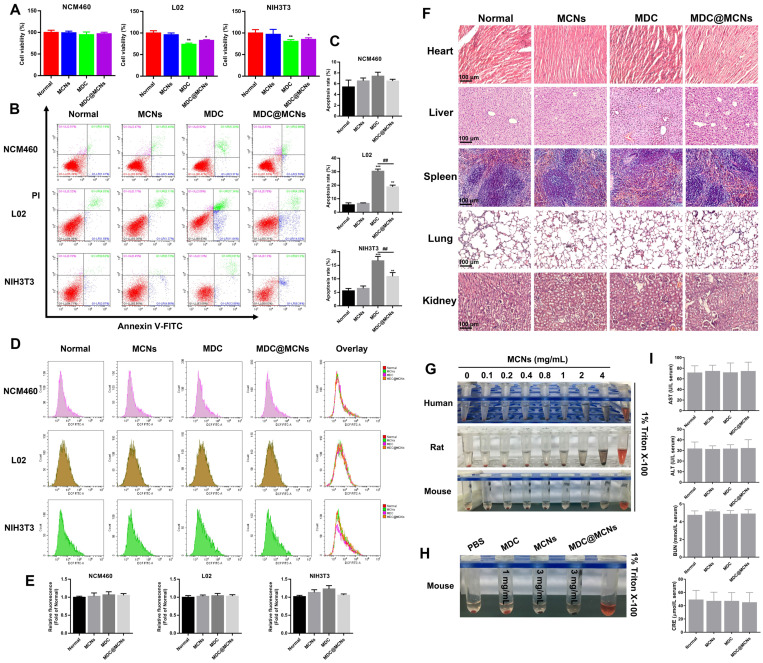
Biocompatibility evaluation of MDC@MCNs *in vitro* and *in vivo*. **(A)** Cell viability of the cells (NCM460, L02 and NIH3T3) treated with various drugs (MCNs, MDC and MDC@MCNs), values are expressed as means ± SEMs, n = 3. **(B)** Apoptosis of the cells induced by various drugs. **(C)** Statistical analysis of apoptosis rate, values are expressed as means ± SEMs, n = 3. **(D)** Fluorescence intensity diagrams reflecting ROS level in NCM460, L02 and NIH3T3 cells induced by various drugs. **(E)** Relative fluorescence intensity statistics, values are expressed as means ± SEMs, n = 3. **(F)** Representative micrographs of H&E staining in heart, liver, spleen, lung and kidney for evaluation of drug toxicology at animal level. **(G)** Evaluation of hemolysis of erythrocytes from three sources (human, rat and mouse) induced by MCNs with different concentration gradients. **(H)** Evaluation of hemolysis for mouse erythrocyte induced by various drugs. **(I)** AST, ALT, BUN and CRE levels in serum of mice treated with the drugs, values are expressed as means ± SEMs, n = 6. *p < 0.05, **p < 0.01, ***p < 0.001 vs Normal group, ^##^*p* < 0.01.

Cell proliferation rate, apoptosis, and ROS production were used as indicators to characterize the potential toxicity of MDC@MCNs on NCM460, L02 human liver and NIH3T3 mouse embryonic fibroblast cells *in vitro*. MCNs at a concentration of 360 μg/mL showed no obvious proliferation inhibition on the above three cell lines. Conversely, 120 μg/mL of MDC suppressed the proliferation of L02 and NIH3T3 but not NCM460 cells. The inhibitory effect of 360 μg/mL (equivalent to 120 μg/mL MDC) of MDC@MCNs on the proliferation of L02 and NIH3T3 cells was not as strong as that of MDC. MDC@MCNs also did not inhibit the proliferation of NCM460 cells (**Figure 4A**). In addition, **Figure 4B-C** demonstrate that none of the drugs induced apoptosis of NCM460 cells. Both MDC and MDC@MCNs promoted apoptosis of L02 and NIH3T3 cells, but the latter drug was less toxic. Surprisingly, the drugs did not cause the three cell lines to produce excessive ROS, compared with the normal group (**Figure 4D-E**). The above results indicated that MCNs, MDC and MDC@MCNs have good biocompatibility to NCM460 cells, and that encapsulation in MCNs may reduce the side effects of MDC.

### Acute toxicology and hemolysis

To assess the toxicity and safety profiles of MDC, MCNs, and MDC@MCNs, mice were treated daily for two weeks. Following treatment, the heart, liver, spleen, lung, and kidney tissues were excised for histopathological analysis. Compared with the normal group, no typical pathological changes were observed in the treated groups (**Figure 4F**). In general, the liver and kidneys are involved in drug metabolism and so indexes of their function are important for evaluating the toxicity of drugs *in vivo*. As shown in **Figure 4I**, serum glutamic oxaloacetic transaminase (AST), glutamic pyruvic transaminase (ALT), blood urea nitrogen (BUN), and creatinine (CRE) levels in each group were normal. These results indicate that administration of five times the therapeutic dose of MDC and MDC@MCNs did not induce significant organ damage. Moreover, hemolysis test results (**Figure 4G**) demonstrate that MCNs of various concentration gradients did not produce hemolysis effects on red blood cells from three sources. More importantly, it was proved that MDC and MDC@MCNs beyond the actual pharmacodynamic dose wouldn't cause erythrocyte hemolysis in mice (**Figure 4H**). In this study, multidimensional evaluation of the safety of this nanoplatform was carried out based on cell proliferation, apoptosis, and ROS production in three cell lines, hemolysis, indicators of liver and kidney function, and H&E staining of major organs. These results suggest that no severe cytotoxicity or systemic toxicity were induced by MCNs, indicating their safety and good biocompatibility. In addition, loading MDC into MCNs not only reduced the toxicity of MDC to L02 and NIH3T3 cells *in vitro*, but also protected it from trypsin damage and promoted its uptake by NCM460 cells 48. In summary, MDC@MCNs have good biocompatibility for further applications.

### MDC@MCNs alleviate the damage of NCM460 cells induced by DSS more effectively

**Figure 5:**
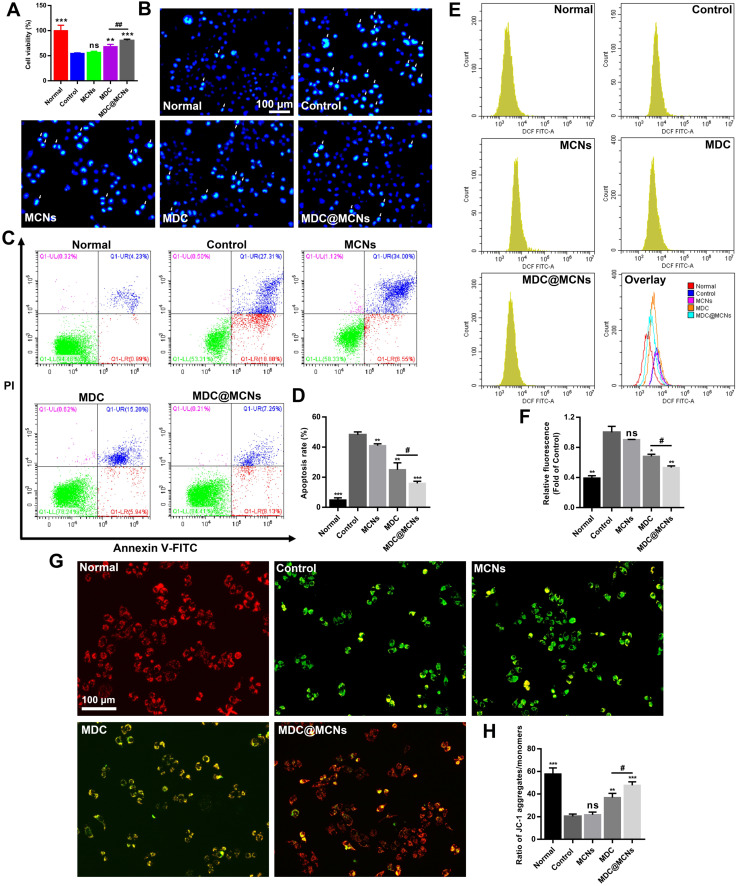
MDC@MCNs alleviate the damage of NCM460 cells induced by DSS more effectively. **(A)** Cell viability of NCM460 cells treated with DSS and drugs, values are expressed as means ± SEMs, n = 3. **(B)** Representative photographs of NCM460 cells nuclei stained with Hoechst 33258 in all the groups. The normal nuclei present ordinary blue, while the nuclei of apoptotic cells are densely or fragmented, densely stained and somewhat whitish. **(C)** Flow cytometry analysis of the effect of drugs on DSS-induced apoptosis in NCM460 cells. **(D)** Quantitative analysis of the percentage of apoptotic cells in each treatment group, values are expressed as means ± SEMs, n = 3. **(E)** Flow cytometry analysis of the effect of drugs on ROS generation in NCM460 cells incubated with DSS. **(F)** The corresponding statistical graph of relative fluorescence intensity, values are expressed as means ± SEMs, n = 3. **(G)-(H)** Change in Mitochondrial membrane potential (MMP) in each group monitored through the ratio of red/green fluorescence. Values are expressed as means ± SEMs, n = 3. ns represents no significant difference, *p < 0.05, **p < 0.01, ***p < 0.001 vs Control group, ^#^*p* < 0.05, ^##^*p* < 0.01.

It is critical for UC treatment to protect intestinal epithelium cells from physical and biochemical stimulation 49. To determine the protective effect of MDC@MCNs on colonic epithelial cell injury *in vitro*, dextran sulphate sodium (DSS) was used to injure NCM460 cells. DSS at an *in vitro* concentration of 10 mg/mL resulted in ~50% cell death. Addition of MCNs did not inhibit the injury, whereas MDC increased cell viability and MDC@MCNs offered even better protection (**Figure 5A**). Apoptosis was also assessed by flow cytometry and Hoechst 33258 staining (**Figure 5B-D**). MDC@MCNs better protected NCM460 cells from DSS-induced apoptosis than MDC. Unexpectedly, MCNs also demonstrated anti-apoptotic effects. To identify the protection mechanism, we assessed ROS production and mitochondrial membrane potential changes, which are closely related to apoptosis 50. Increased ROS production inducing apoptosis was verified in **Figure 5E-F**. MDC reduced ROS production and MDC@MCNs exhibited even better action. To assess mitochondrial membrane potential changes, we used the fluorescent probe JC-1, which aggregates in the mitochondrial matrix to form a polymer (J-aggregates) with red fluorescence representing high mitochondrial membrane potential. At low mitochondrial membrane potential, JC-1 is monomeric with green fluorescence. As shown in **Figure 5G-H**, DSS significantly decreased the mitochondrial membrane potential of NCM460 cells. MDC@MCNs significantly curbed the decline in mitochondrial membrane potential more effectively than MDC. In summary, DSS increased ROS production and decreased mitochondrial membrane potential in NCM460 cells, resulting in apoptosis. DSS-induced injury was improved by MDC@MCNs treatment via effective control of the excessive ROS production and decreased mitochondrial membrane potential. Although MDC showed therapeutic effects, it was far less effective than MDC@MCNs, which may be attributed to its weaker uptake by NCM460 cells.

### MDC@MCNs significantly improve survival status and colonic injury in UC mice

**Figure 6:**
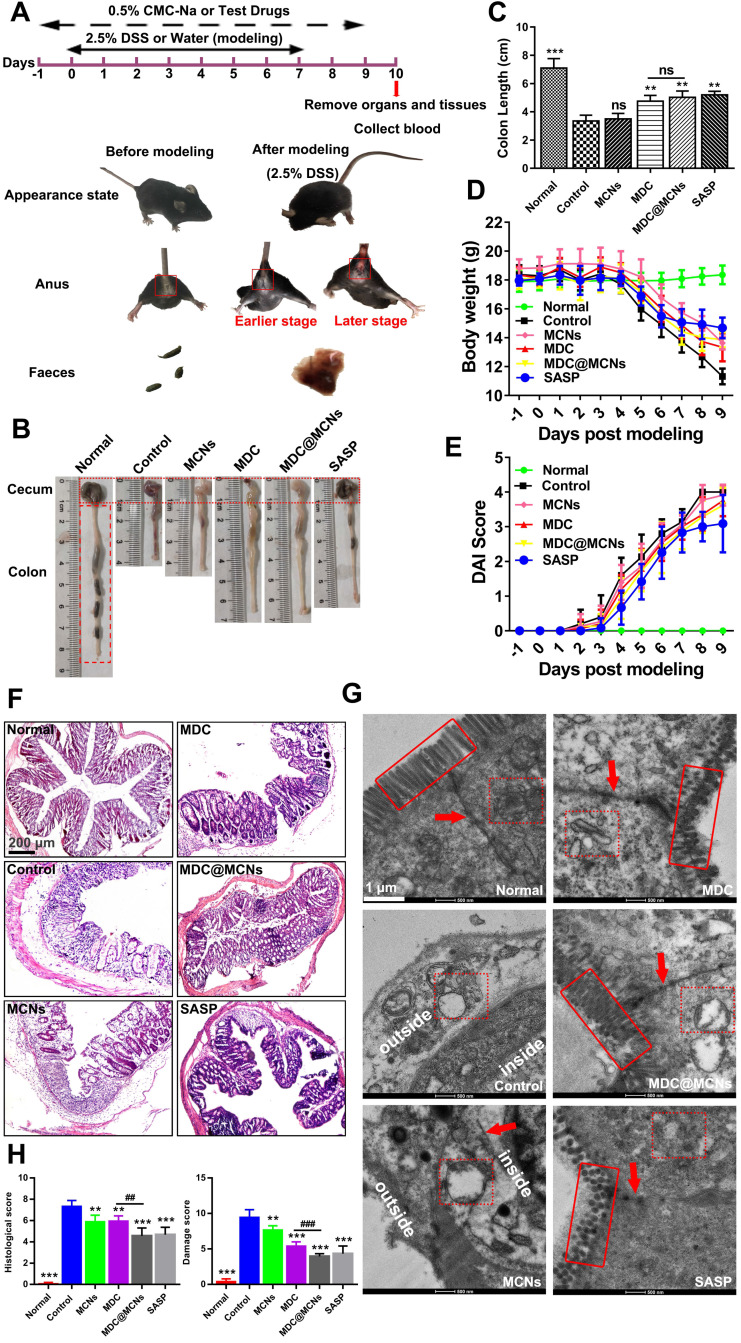
The positive effects of MDC@MCNs on survival state, colon length, body weight, DAI, pathological structure damage of colon in DSS-treated mice. **(A)** The experimental design and differences in appearance, perianal area and feces before and after mouse modeling. **(B)** Typical colonic segment appearance of each group. **(C)** Statistical graph of colon length in each group.** (D-E)** Changes in body weight and DAI score of mice during the trial period. **(F-G)** Representative H&E staining photomicrographs and TEM ultrastructure images of colon sections from each experimental group. **(H)** Histological damage score based on the results of H&E staining and TEM in all the groups. Values are expressed as means ± SEMs, Normal group (n = 10), Control group (n = 15), MCNs group (n = 15), MDC group (n = 15), MDC@MCNs (n = 15), SASP group (n = 12). ns represents no significant difference, **p < 0.01, ***p < 0.001 vs Control group, ^##^*p* < 0.01, ^###^*p* < 0.001.

UC with complex and unclear etiology has been widely studied in recent years. Acute UC causes body weight loss, diarrhea, fecal bleeding and even death frequently 51. A mouse model of UC was successfully established through free drinking of sterilized water containing 2.5% DSS for 8 days. The weight of the model mice was significantly reduced and the phenomenon of hematochezia occurred with poor mental state (**Figure 6A and D**). The shorten colon segment from the cecum to rectum indirectly indicated the existence of colon injury. **Figure 6B-C** shows that both MDC and MDC@MCNs restricted the shortening of the colon to a similar extent as the positive drug salicylazosulfapyridine (SASP). Additionally, MDC and MDC@MCNs reduced weight loss and disease activity index (DAI) in mice (**Figure 6D-E**). H&E staining and TEM results (**Figure 6F-H**) further demonstrate the beneficial effects of these drugs for preventing the histopathological and microstructure damage to the colon caused by UC. Compared with the normal group, the colonic mucosal epithelial injury in the control group was extremely severe with ulcer formation, inflammatory cell infiltration, crypt deformation or even loss, decreased goblet cells, and disappeared colonic gland. MCNs prevented some loss of the crypt. MDC@MCNs remarkably recovered colonic mucosal injury of the crypt to almost normal and significantly reduced inflammatory cell infiltration, proving to be more effective than MDC and even SASP (**Figure 6F**). In **Figure 6G**, the microvilli of the normal group are shown to be well-distributed and the colonic epithelial cells have a compact arrangement and are in good condition, forming complete tight junctions. In contrast, cell necrosis with exudative intracellular material occurred in the control group, accompanied by loss of microvilli and complete destruction of tight junctions. Although MCNs failed to prevent the loss of microvilli, it prevented leakage of intracellular substances and protected the integrity of the tight junctions. MDC@MCNs provided better effects than MDC and SASP as they were not only capable of maintaining the integrity of intracellular organelles and tight junctions, but also prevented microvilli from being destroyed. The degree of colon damage is judged more directly in **Figure 6H**. In summary, all of the above results demonstrate that MDC@MCNs significantly improved survival status and colonic injury in UC mice.

### MDC@MCNs markedly inhibit inflammation and oxidative stress in UC mice

Although the pathogenesis of UC remains unclear, immune and inflammatory responses and oxidative stress are absolutely involved and play vital roles. Overproduction of pro-inflammatory cytokines and indicators such as TNF-α, IL-6, IL-1β, IFN-γ, NF-κB p65, COX-2, and INOS is very obvious with progression of UC 52, 53. The imbalance in immune responses induced by UC usually stimulates the T helper cell 17 (Th17) population to induce inflammation, while decreasing T regulatory cells (Treg) to further promote Th17 activity 54. Therefore, increasing studies have focused on the roles of IL-10 (Treg secretion) and IL-17 (Th17 secretion) in UC, which result in extensive immunosuppression and immune inflammation, respectively 55, 56. DAO is an enzyme specifically located at the apical end of mature villous cells that catalyzes the oxidation of diamines such as histamine, putrescine, and cadaverine, reflecting the integrity and maturity of the intestinal mucosa 57. Damaged mucosal cells release DAO, which increases its serum concentration. Owing to the stable activity of DAO in the blood 58, the concentration of DAO in the blood may reflect the damage and restoration of the intestinal cavity in a timely manner 59. In addition, DSS often induces colonic oxidative stress, indicated by oxidation and antioxidant markers including MDA level and MPO, CAT, SOD, and GSH-Px activities 60.

**Figure 7:**
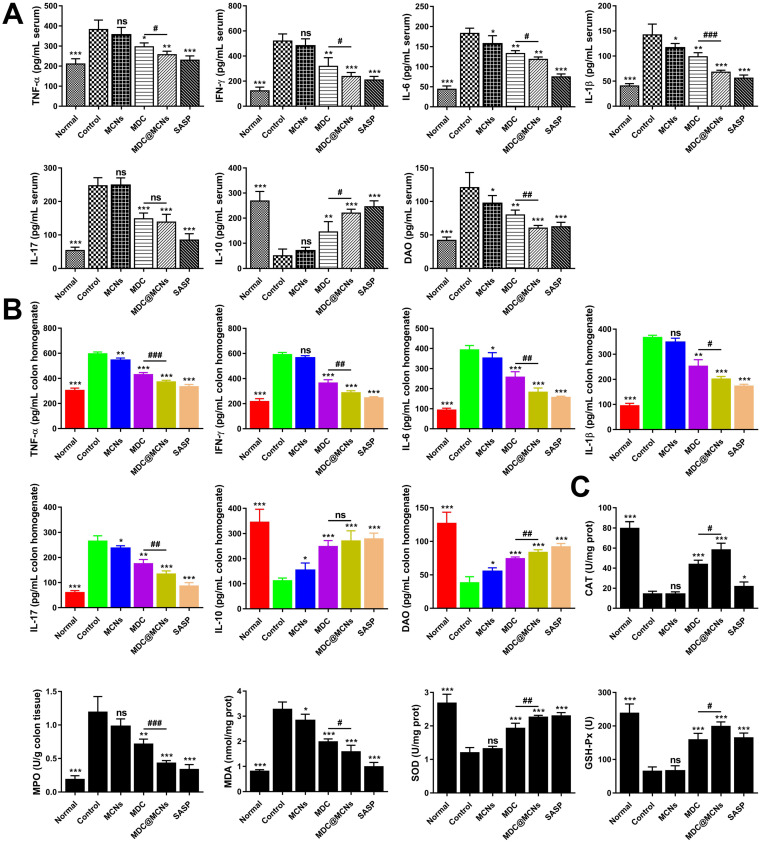
MDC@MCNs markedly inhibit inflammation and oxidative stress in UC mice. **(A)-(B)** The contents of inflammation-related cytokines including TNF-α, IFN-γ, IL-6, IL-1β, IL-17, IL-10 and the intestinal barrier damage indicator DAO in serum and colon tissues of all the groups. **(C)** The activities of oxidase MPO, antioxidant enzyme CAT, SOD and GSH-Px as well as the content of MDA in colon tissues of six groups. Values are represented as means ± SEMs, n = 6. ns represents no significant difference, *p < 0.05, **p < 0.01, ***p < 0.001 vs Control group, ^#^*p* < 0.05, ^##^*p* < 0.01, ^###^*p* < 0.001.

**Figure 8:**
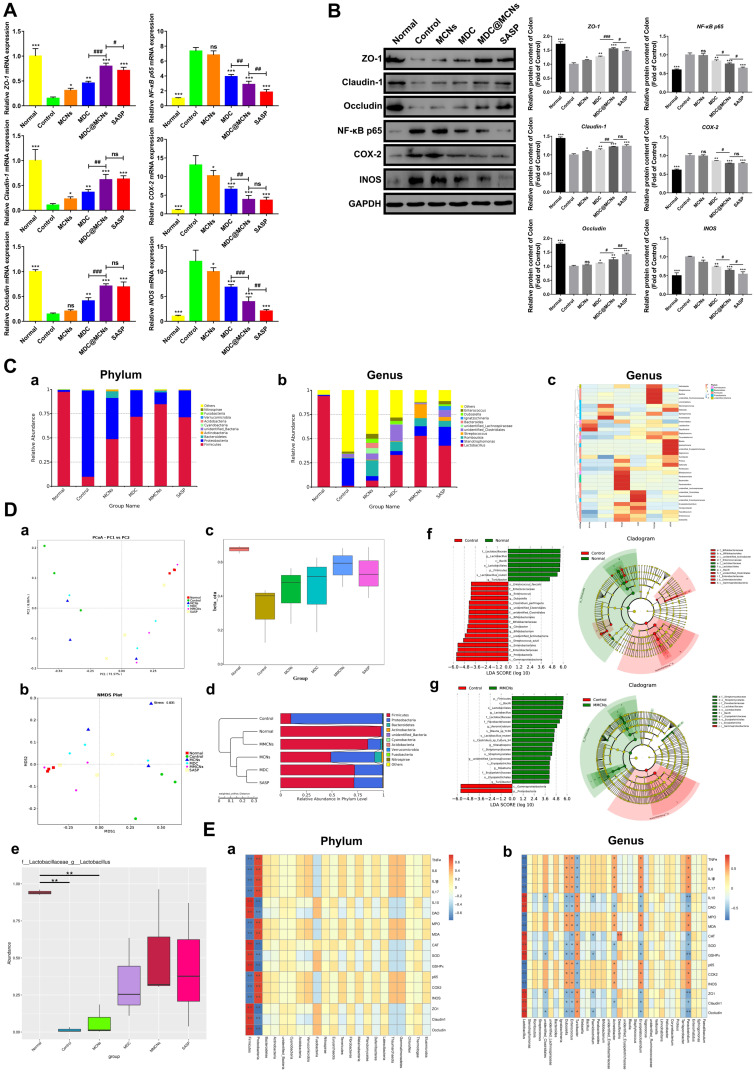
MDC@MCNs significantly reduce colonic inflammation and preserve tight junctions of colon tissues as well as ameliorate intestinal flora imbalance in UC mice. **(A)** Relative mRNA expression of ZO-1, Claudin-1, Occludin, NF-κB p65, COX-2 and INOS in colon tissues of six groups. **(B)** Western blotting analyses of these proteins expression in relative to GAPDH in the colon tissues of all the groups, consisting of representative strips and corresponding statistical graphs. Values are represented as means ± SEMs, n = 6. ns means no significant difference, *p < 0.05, **p < 0.01, ***p < 0.001 vs Control group, ^#^*p* < 0.05, ^##^*p* < 0.01, ^###^*p* < 0.001. **(C) a-b** Relative abundance of top 10 species at phylum and genus level in six groups. **(C) c** Clustering thermogram of abundance of top 35 species at genus level, the horizontal axis and the vertical axis represent groups and species annotation information, respectively. The left side is the species cluster tree, and the color of the rectangular lattice corresponds to the z value obtained after the normalization of the relative abundance of the species. **(D) a-d** Principal co-ordinates analysis (PCoA), non-metric multi-dimensional scaling (NMDS), beta diversity index and weighted unifrac-based UPGMA clustering tree among different groups. PCoA, NMDS and beta diversity index are also based on weighted unifrac. **(D) e** MetaStat analysis used to identify species with significant difference among groups at genus level. **(D) f-g** Linear discriminant analysis (LDA) plots highlighting significantly different characteristic taxons between microbiota of normal and control group as well as between control and MDC@MCNs group, only those taxa with a value of LDA score more than 4 are considered. Taxonomic cladogram of bacterial samples in all the groups. **(E) a-b** Spearman correlation analysis between biochemical indicators and species at phylum and genus levels. Spearman correlation coefficient r ranges from -1 to 1, r < 0 is negative correlation, r > 0 is positive correlation. *p < 0.05, **p < 0.01.

To assess the impact of MDC@MCNs on inflammation in UC mice, TNF-α, IFN-γ, IL-6, IL-1β, IL-17, IL-10, and DAO levels were detected in serum and colon tissues (**Figure 7A-B**). DSS treatment (control group) increased TNF-α, IFN-γ, IL-6, IL-1β, IL-17, and DAO and decreased IL-10 in serum. MDC, MDC@MCNs, and SASP reversed these changes to varying degrees. Overall, MDC@MCNs and SASP better ameliorated the pro-inflammatory factors (TNF-α, IFN-γ, IL-6, IL-1β, IL-17, and DAO) and the anti-inflammatory factor (IL-10) than MDC. Additionally, the drugs had similar effects on these inflammatory cytokines (except DAO) in colon tissues. The DSS-induced decrease in colonic levels of DAO was markedly impeded by treatment with MCNs, MDC, MDC@MCNs, and SASP. In addition, MCNs suppressed the production of IL-6, IL-1β, and DAO in serum, and decreased the levels of TNF-α, IL-6, IL-17 and increased IL-10 and DAO in colon tissues. The effect of MDC@MCNs on oxidative stress in UC mice was investigated by determining MDA levels and the activities of MPO, CAT, SOD, and GSH-Px in colon tissues. As shown in **Figure 7C**, MDC, MDC@MCNs, and SASP greatly promoted the activities of antioxidant enzymes including CAT, SOD, and GSH-Px as compared with the control group. Furthermore, the drugs largely depressed MPO activity and MDA levels. In particular, MDC and MDC@MCNs better improved CAT activity than SASP. NF-κB p65, COX-2, and INOS mRNA and protein expressions in colon tissues were also determined (**Figure 8A-B**). The results indicate that MDC, MDC@MCNs, and SASP reduced the mRNA and protein expressions of NF-κB p65, COX-2, and INOS in colitis tissues, while MCNs slightly suppressed the mRNA expressions of COX-2 and INOS as well as the protein expression of INOS. Based on these results, it is concluded that MDC@MCNs markedly inhibited the inflammation and oxidative stress caused by UC and favorably regulated immune disorder. The effect of MDC@MCNs on inflammation and oxidative stress was much stronger than that of MDC, and was advantages compared to SASP in some respects such as CAT activity improvement.

### MDC@MCNs availably maintain colonic tight junctions in UC mice

Intestinal barrier injury is recognized as one of the main reasons for UC 61. The normal intestinal barrier formed by epithelial cells and the junctional complex, which includes tight junctions, plays an important role in mucosal immunity, inflammation, and the invasion of harmful substances and microbiota. The tight junctions mainly consisting of ZO-1, claudin, and occludin are the principal determinants of mucosal permeability 62. The intestinal epithelium is covered with a dense layer of mucus to prevent translocation of the gut microbiota into underlying tissues 63. The negative alterations to tight junctions and colonic mucus layer integrity and permeability occurring in UC may lead to gut bacterial encroachment that can eventually cause inflammation and further aggravate infection 64, 65.

Given their importance in UC, we next sought to clarify the effects of MDC@MCNs on the mRNA and protein expressions of tight junctions in colon tissues after DSS exposure. As expected, down-regulation of tight junction mRNA and protein occurred in the colon tissues of UC mice, while MDC, MDC@MCNs, and SASP enhanced the mRNA and protein levels of ZO-1, claudin-1, and occludin (**Figure 8A-B**). MDC@MCNs and SASP, more than MDC, considerably regulated expression of tight junction protein. Surprisingly, MCNs also upregulated the expressions of ZO-1 and claudin-1. Two methods confirmed the reliable result that MDC@MCNs maintained tight junctions in the colons of mice with UC, which played a vital role in the restoration of the colonic mucosal barrier to prevent further deterioration of UC. In theory, MCNs should not have biological activity in UC treatment, but the results demonstrate that they could improve colon injury and inflammatory and oxidative responses in UC mice to some extent, which may be related to filling the injured mucus layer and intercellular space through physical adsorption, preventing the loss of tight junction protein and maintaining the mucosal barrier (**Figure 1C**). Based on this unexpected effect of MCNs, MDC@MCNs showed a great advantage over MDC in maintaining the intestinal mucosal barrier and provided a better therapeutic effect on UC.

### MDC@MCNs ameliorate intestinal flora imbalance in UC mice

An increasing body of evidence has shown that intestinal flora can impact the occurrence of UC 66-70 because of its induction of persistent intestinal inflammation and intestinal barrier injury. Conversely, changes to the intestinal barrier and inflammation and oxidative stress are also able to affect the composition of intestinal flora 71. To clarify the relationship between the pathogenesis of UC and the flora and drug action, 16s rDNA sequencing was carried out. As shown in **Figure 8C a-c**, the composition of intestinal flora in UC mice (control group) was significantly different from that of other groups. In the control group, *Proteobacteria* occupied the main components at the phylum level while *Firmicutes* decreased sharply, which is consistent with previous studies 72, 73. At the genus level,* Lactobacillus*, a common beneficial bacteria 74, and *Desulfovibrio* were dominant in the normal group, while *Stenotrophomonas* and* Acinetobacter* were dominant in the control group. As expected, the drugs inhibited DSS-induced reduction of *Lactobacillus*. MCNs, MDC, MDC@MCNs, and SASP also improved the species composition to be closer to that of the normal group than the control group. MCNs vastly increased the relative abundances of *Proteus*, *Romboutsia*, *Bifidobacterium*, *Parabacteroides*, *Bacteroides*, *Paraclostridium*, unidentified* Lachnospiraceae*, *Erysipelatoclostridium*, *Enterococcus*, and *Dubosiella*. The relative abundances of unidentified* Clostridiales*, *Paeniclostridium*, unidentified* Enterobacteriaceae*, *Erysipelatoclostridium*, *Terrisporobacter*, and *Faecalibaculum* in the MDC group was also increased. Most importantly and interestingly, MDC@MCNs (MMCNs) did not simply superimpose the regulation effects of MDC and MCNs on the bacteria abundances but greatly increased the abundances of *Helicobacter*, *Streptococcus*, *Bacillus*, unidentified* Ruminococcaceae*, *Limnohabitans*, *Sphingomonas*, *Staphylococcus*, and *Corynebacterium*. SASP strongly increased the abundances of *Klebsiella*, *Staphylococcus*, *Blautia*, *Ignatzschineria*, unidentified* Erysipelotrichaceae*, *Vagococcus*, *Turicibacter*, *Proteus*, and *Veillonella*. Beta diversity analyses consisting of principal co-ordinates analysis (PCoA), non-metric multi-dimensional scaling (NMDS), beta diversity index, and UPGMA clustering tree were used to distinguish similarity differences among different groups (**Figure 8D**). Both PCoA and NMDS analyses reflect the differences between samples or groups by the distance between points. The distance between the MDC@MCNs group and the normal group samples was the smallest, followed by the SASP group, then the MDC group, the MCNs group, and finally the control group, meaning that the relative strength in intestinal flora regulation ability is MDC@MCNs > SASP > MDC > MCNs (**Figure 8D a-b**). PCoA and NMDS analysis results are supported by a box diagram of beta diversity inter-group difference analysis (**Figure 8D c**). As the UPGMA clustering tree shows (**Figure 8D d**), the community structure of the MDC@MCNs group is most similar to that of the normal group, illustrating its considerable positive effects on the intestinal flora. Although MCNs, MDC, and SASP played regulatory roles, their effects were not as strong as those of MDC@MCNs. Further, MetaStat was performed to further identify species with obvious differences between groups. *Lactobacillus* was found to be the maximal differential species between groups at the genus level (**Figure 8D e**), which validated the previous species abundance results. We further applied the linear discriminant analysis effect size (LEfSe) analytic method to discriminate differentially abundant bacterial taxa among these groups (only those taxa with a log [LDA] score > 4 were ultimately considered). The statistical results of LEfSe include three parts: histogram of LDA value distribution, evolutionary cladogram (phylogenetic distribution), and abundance comparison map of biomarker with statistical differences among groups. **Figure 8D f** clearly shows that the levels of *Gammaproteobacteria*, *Proteobacteria*, *Enterobacteriaceae*, *Enterobacterales*, *Streptococcus azizii*, unidentified* Actinobacteria*, *Bifidobacterium*, *Citrobacter*, *Bifidobacteriaceae*, *Bifidobacteriales*, unidentified* Clostridiales*, unidentified* Clostridiales*, *Clostridium perfringens*, *Dubpsiella*, *Enterococcus*, *Enterococcaceae*, and *Enterococcus faecalis* were obviously increased in the colonic microbiota with DSS treatment compared with the normal group, while *Lactobacillaceae*, *Lactobacillus*, *Bacilli*, *Lactobacillales*, *Firmicutes*, *Lactobacillus reuteri*, and *Turicibacter* were found to be the absolutely dominant microbiota in the normal group. On the other hand, when compared with the MDC@MCNs group (**Figure 8D g**), *Gammaproteobacteria* and *Proteobacteria* represented the most influential biomarkers of the control group, and the following microbiota increased in the MDC@MCNs group: *Firmicutes*, *Bacilli*, *Lactobacillales*, *Lactobacillus, Lactobacillaceae*, *Flavobacteriaceae*, *Aeromicrobium*, *Blautia sp. YL58*,* Lactobacillus reuteri*, *Clostridium sp. Culture 54*, *Kitasatospora*, *Streptomycetaceae*, *Streptomycetales*, unidentified* Bachnospiraceae*, *Erysipelotrichia*, *Roseburia*, *Erysipelotrichaceae*, *Eirysipelotrichales*, and *Turicibacter*. On the whole, MDC@MCNs transformed the intestinal flora disorder induced by DSS to normal.

In order to further investigate the relationship between intestinal flora and all colonic biochemical indicators including TNF-α, IL-6, IL-1β, IL-17, IL-10, DAO, MPO, MDA, CAT, SOD, GSH-Px, NF-κB p65, COX-2, INOS, ZO-1, claudin-1, and occludin, a Spearman correlation analysis was conducted. As shown in **Figure 8E a**, *Firmicutes* and *Proteobacteria* at the phylum level showed an obvious opposite relationship. Increased levels of indicators in favor of UC improvement such as ZO-1, claudin-1, occludin, CAT, SOD, GSH-Px, IL-10, and DAO in colon tissues were dramatically associated with enhanced abundance of *Firmicutes* and reduction in *Proteobacteria*. In addition, these indicators exhibited positive correlation with *Lactobacillus* and* Turicibacter* at the genus level, which may depress the levels of TNF-α, IL-6, IL-1β, IL-17, MPO, MDA, NF-κB p65, COX-2, and INOS. *Enterococcus*, *Dubosiella*, *Acinetobacter*, *Erysipelatoclostridium*, and *Paraclostridium* may further increase inflammation and oxidative stress as well as destroy tight junctions by increasing the levels of TNF-α, IL-6, IL-1β, IL-17, MPO, MDA, NF-κB p65, COX-2, and INOS and decreasing the levels of ZO-1, claudin-1, occludin, CAT, SOD, GSH-Px, IL-10, and DAO in colon tissues (**Figure 8E b**). In summary, MDC@MCNs regulate intestinal flora, which may be attributed to or conducive to effective anti-inflammation and antioxidation effects and maintenance of tight junctions. There are close relationships between intestinal flora, inflammatory and oxidative responses, and intestinal barrier that decisively impact UC progression.

### MDC@MCNs are retained in intestines due to intestinal adhesion and aggregation of MCNs

**Figure 9:**
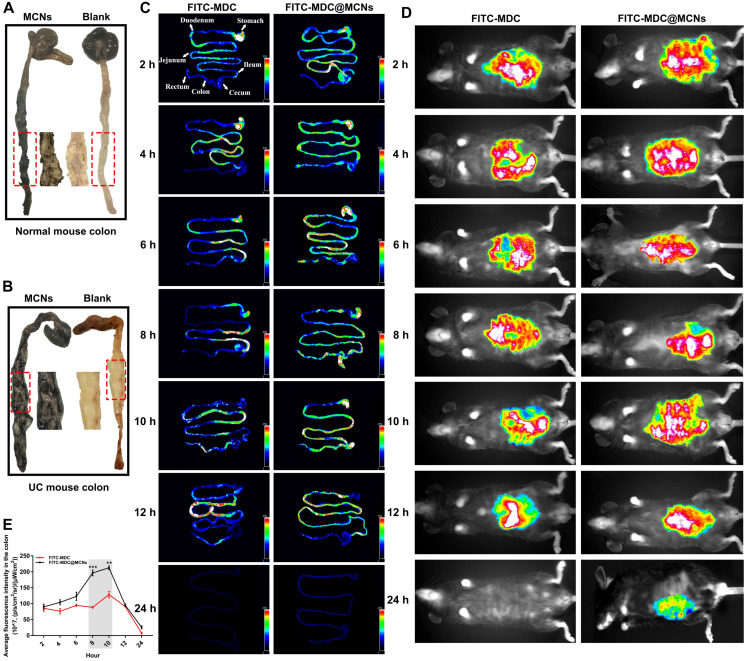
MDC@MCNs are retained in intestines due to intestinal adhesion and aggregation of MCNs.** (A)-(B)** Bioadhesion of MCNs assessed by black staining of the colon tissues following administration of MCNs in normal and UC mice. The intestinal segments were fully washed with saline before photographing. **(C)** Typical photographs of biological distribution of FITC-MDC and FITC-MDC@MCNs in digestive tract of normal mice at different time points after oral administration. **(D)** Live whole-body fluorescent imaging of mice. **(E)** Quantitative analysis of fluorescence intensity in the colon showing payload bioavailability in the colon. Values are represented as means ± SEMs, n = 3. **p < 0.01, ***p < 0.001 vs FITC-MDC group.

Black staining of the colons of normal and UC mice after administration of MCNs is shown in **Figure 9A-B**. The normal colon in blank group appears in a regular state with clear microvessels and mucosa, whereas there is congestion with ulcer formation in colon of UC. When MCNs were orally administrated, the colons of mice with or without UC were significantly stained black after 4 h. Although the intestinal segment was carefully rinsed with saline, the black staining was still strongly retained, indicating that MCNs had firmly adhered to the epithelial layer. MCNs were more likely to adhere to the diseased colon in UC than to normal colon. In the area of colitis, decreased mucin sulfurization with increased cationic protein expression promotes a positive surface charge 75, 76, enhancing coulombic interactions between MCNs and colon surfaces. It has also been reported 77 that the range of pH in the normal colon is ~6.8-7.2, and this is decreased to some extent in the colons of mice with UC. These reported findings provide a theoretical basis for the accumulation and drug release of MDC@MCNs in the colon of UC mice. The biodistributions of FITC-MDC and FITC-MDC@MCNs in the gastrointestinal tract of mice were visually investigated using a fluorescence imaging system (**Figure 9C-E**). After oral administration, the fluorescence intensities of FITC-MDC and FITC-MDC@MCNs in the stomach decreased over time and accumulation in the intestinal tract increased. At 2 h post administration, the fluorescence intensity of FITC-MDC was mainly concentrated in the stomach, whereas FITC-MDC@MCNs were also found throughout the small intestine and especially in the ileum. Over time (4-24 h), the fluorescence intensity of FITC-MDC in the intestinal tract gradually weakened and disappeared, and FITC-MDC barely accumulated in the colon. In contrast, FITC-MDC@MCNs were able to remain confirmedly in the intestine and stomach for a long time (24 h as maximum experimental time point).

In summary, the excellent intestinal adhesion of MCNs allowed MDC@MCNs to be transported for a longer time than MDC, which would be beneficial to subsequent transepithelial absorption and would enhance bioavailability. Moreover, intestinal adhesion of MCNs is even more prominent in UC mice than healthy mice. Most importantly, the achieved long-term retention of MDC@MCNs in colon tissue provides a practical basis for the targeted treatment of UC.

## Conclusions

MDC@MCNs are a rationally designed colon-accumulating nanoplatform for effective MDC delivery with good biocompatibility. Oral administration of MDC@MCNs significantly ameliorated UC by inhibiting inflammation and oxidative stress, maintaining intestinal mucosal barrier, and regulating intestinal flora, with an efficacy and safety superior to that of MDC. Therefore, MDC@MCNs may provide a novel precise and safe therapeutic strategy for UC.

## Experimental section

### Materials

*Musca domestica* cecropin (MDC, molecular weight: 4301.59 Da) with purity of 97.15% was chemically synthesized by conventional Fmoc solid-phase synthetic strategy (Beijing Scilight Biotechnology Ltd., Beijing, China) 78. The MDC decorated by fluorescin isothiocyanate (FITC-MDC) was also provided by the above company. Sulfasalazine enteric-coated tablets (SASP) was produced by Shanghai Forward Pharmaceutical Co,. Ltd. Dextran sulfate sodium (DSS, molecular weight: 36000-50000 Da) was purchased from MP Biomedicals (Santa Ana, CA, USA). F-127 was acquired from Sigma-Aldrich (St. Louis, MO, USA). Phloroglucinol, formaldehyde and dimethyl sulfoxide (DMSO) were obtained from Aladdin (Shanghai, China). All other chemical reagents were standard commercial products with analytical reagent grade.

### Synthesis of mesoporous carbon nanospheres (MCNs)

In a typical experiment, 6 mL of hydrochloric acid (w%, 36%), 0.4 g of F127 and 0.24 g of phloroglucinol were dissolved in 40 mL of deionized water with magnetic stirring at 25°C. Subsequently, 0.14 mL of formaldehyde solution was slowly added into the mixed solution above for 2 h reaction followed by two processes: kept at 40°C for 4 h and at 60°C for 24 h. After centrifugation, the sample was washed with deionized water in the centrifuge tube for three times, drying in the oven at 45°C. Finally, mesoporous carbon nanospheres (MCNs) were obtained by carbonization at 600°C in nitrogen atmosphere.

### Preparation of MDC/FITC-MDC-containing mesoporous carbon nanoparticles (MDC@MCNs/FITC-MDC@MCNs)

In brief, 10 mg MDC/FITC-MDC and 10 mg MCNs were added to 10 mL bidistilled water and stirred for 4 h. MDC/FITC-MDC not loaded into the holes of MCNs was removed by rotating centrifugation at a speed of 2000 r/min for 5 min. The above removed supernatant containing MDC/FITC-MDC was preserved to be measured. Then the rest mixture was transferred to an evaporating basin to be evaporated until becoming dry completely. The resulting products MDC/FITC-MDC-containing mesoporous carbon nanoparticles were obtained defined as MDC@MCNs/FITC-MDC@MCNs and weighed.

### Determination of loading content of MDC/FITC-MDC into MCNs

To determine the drug loading rate of MDC@MCNs, the BCA method for determination of MDC/FITC-MDC content was established, which was based on the instructions of Enhanced BCA Protein Assay Kit (Beyotime, Shanghai, China). The only difference was that MDC/FITC with different concentration gradients (0.025, 0.05, 0.1, 0.2, 0.3, 0.4, 0.5 and 1 mg/mL) replaced standard protein as standard reference substance. The formula for calculating drug loading rate was as follows: 
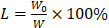
; W_0_ and W represented the content of MDC/FITC-MDC loaded into MCNs and weight of MDC@MCNs/FITC-MDC@MCNs, respectively. It's remarkable that the content of MDC loaded into MCNs was acquired indirectly by deduction of MDC not loaded in MCNs from 10 mg MDC.

### *In vitro* release analysis of MDC

Continuous release of MDC from MDC@MCNs of 10 mg was performed with uniform magnetic stirring at 37°C for 6 h, including two stages as following: the release was accomplished in 10 mL simulated artificial gastric fluid (pH 1.2, USP) for 2 h at first stage and MDC@MCNs were transferred into 10 mL intestinal fluid (pH 6.8, USP) for 4 h at second stage. In order to determine the release of MDC, the supernatant of 1 mL was obtained after centrifugation (3500 g/5 min) at particular points in time (Stage1: 30, 60, 90, 120 min; Stage 2: 150, 210, 240, 300, 360 min). Then fresh medium of 1 mL was immediately added to the release system before continuing the simulated release. The cumulative release rate was obtained by using the following formula: 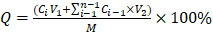. C_i_ and C_i-1_ were the concentrations of MDC in supernatant (sampling times: i and i-1) determined by the BCA method as previously described. V_1_ and V_2_ indicated the invariant volume of release medium (10 mL) and sampling (1 mL). M was the initial amount of MDC in release process equal to the content of MDC in 10 mg MDC@MCNs. Finally, sampling time and cumulative release rate were used as the horizontal and vertical coordinates to plot the release curve.

### Characterization of MCNs and MDC@MCNs

Nitrogen adsorption measurements were performed at 77 K on a Belsorp-miniII (MicrotracBEL, Osaka, Japan). The X-ray diffraction (XRD) patterns were recorded by using a Bruker D8 Advance X-ray diffractometer (Karlsruhe, Germany) with Cu Kα radiation (40 kV, 40 mA). Transmission electron microscopy (TEM) was carried out on a FEI Tecnai G2 F30 (Oregon, USA) operated at 300 kV. The particle size and zeta potential of the MCNs and MDC@MCNs in PBS solution (pH 6.8) were measured at 25°C on Zetasizer Nano-ZS90 (Malvern, UK).

### Stability determination of MDC@MCNs after trypsinase treatment

To investigate whether MCNs could protect MDC activity from trypsin-induced damage, the oxford cup antibacterial experiments were conducted to determine the changes of bacteriostatic activity of MDC and MDC@MCNs before and after trypsinase treatment *in vitro* using *Salmonella typhimurium* as indicator bacteria. Luria-Bertani (LB) agar medium was heated to be liquid state (100 mL) and then 100 μL of *Salmonella typhimurium* suspension (1×10^9^-5×10^9^ CFU/mL) in logarithmic phase was diluted into the liquid medium above. The medium containing *Salmonella typhimurium* was poured onto sterilized petri dishes to allow solidification in about 5 min. Immediately, oxford cup was put on the surface of solidified LB medium and gently pressured to closely contact with the LB medium. In the end, 100 μL of MDC (1 mg/mL), MCNs (3 mg/mL) and MDC@MCNs (3 mg/mL) treated with or without trypsin were added into the oxford cup. The petri dishes were incubated at 37°C for 10 h and the antagonistic activity was estimated by the size of the bacteriostatic ring imaged using a Molecular Imager Gel Doc XR+ System (Bio-Rad, CA, USA). The experiment was repeated three times under the same conditions to afford the error estimates.

### Cell culture conditions

NCM460, L02, and NIH3T3 cells were maintained in Dulbecco's Modified Eagle's Medium (DMEM, Gibco, NY, USA). All cell lines were supplemented with 10% fetal bovine serum (FBS, Gibco) and 1% penicillin/streptomycin (Invitrogen, CA, USA) and maintained at 37°C in a humidified atmosphere with 5% CO2 and 95% air.

### Cellular uptake assay

The NCM460 cells (2 × 10^5^ cells per well) were seeded in a six-well plate and cultured for 24 h until being adherent state. Then the medium was removed and PBS was added to each well for washing three times. Subsequently, the 2 mL medium containing FITC-MDC@MCNs (360 μg/mL) and FITC-MDC (120 μg/mL) were added to the plates. After 2 h of treatment, the cells were fixed with 4% paraformaldehyde solution, and DAPI (5 μg/mL) was used to stain their nuclei. Eventually, images were captured using a fluorescence microscope (Zeiss, Oberkochen, Germany). Fluorescence intensity was analyzed to show statistical differences by using Image J software.

### MTT assay

Briefly, cells (NIH3T3, L02 and NCM460) in good condition were digested and diluted to 1 × 10^5^ cells/mL with culture solution. The 100 µL of cell suspension was seeded onto a 96-well plate and incubated for 24 h. In the section of evaluation for cytotoxicology, the cytotoxic activities of MCNs (360 μg/mL), MDC (120 μg/mL) and MDC@MCNs (360 μg/mL) on the three cell types for 24 h were assessed by the MTT assay respectively. Moreover, MTT assay was also used to investigate the effect of drugs above on DSS-induced NCM460 cells injury *in vitro* model 79. NCM460 cells were plated and grown to 80% confluence prior to treatment with drugs. After 12 h, the NCM460 cells were exposed to DSS of 10 mg/mL for an additional 12 h. Next, the cells were exposed to MTT reagent (5 mg/mL, 20 µL) for another 4 h. After discarding the medium, 100 µL of DMSO was added to each well, followed by shaking for 5 min to dissolve the formazan crystals. The absorbance at 570 nm was determined using a microplate reader (BioTek SynergyH1, Vermont, USA). The cell viability with data from three parallel wells was calculated.

### Cell apoptosis and ROS detection by flow cytometry

Apoptosis was determined using Annexin V-FITC Apoptosis Detection Kit (Beyotime, Shanghai, China) in accordance with the manufacturer's instructions. The cells were harvested and washed with cold PBS solution. Subsequently, the cells were resuspended in 195 µL of binding buffer and incubated with 5 µL of Annexin V-FITC and 10 µL of propidium iodide (PI) in the dark environment at 25°C for 15 min. Reactive Oxygen Species Assay Kit (Beyotime, Shanghai, China) was used to measure intracellular changes in ROS generation. The cells were stained with 10 μM DCFH-DA at 37°C for 30 min followed by washing with PBS. Finally, the prepared samples were immediately detected using a CytoFLEX flow cytometer (Beckman Coulter, Miami, USA) and analyzed by the matched CytExpert software program.

### Hoechst 33258 staining

Hoechst 33258 staining was performed according to the protocol of Hoechst Staining Kit (Beyotime, Shanghai, China). The NCM460 cells were stained with Hoechst 33258 staining solution in the cell incubator without light for 20 min before being washed three times with PBS. The morphology of cell nuclei was obtained under the fluorescence microscope.

### Mitochondrial membrane potentials assay

JC-1 probe was employed to measure mitochondrial depolarization in NCM460 cells. All operations were performed in accordance with the Mitochondrial membrane potential assay kit with JC-1 (Beyotime, Shanghai, China) instructions. Briefly, the cells cultured in twelve-well plates after indicated treatments were incubated with an equal volume of JC-1 staining solution at 37°C for 20 min in dark and rinsed slightly twice with PBS. Mitochondrial membrane potentials were monitored by determining the relative amounts of dual emissions from mitochondrial JC-1 monomers (excitation of 514 nm and emission of 529 nm) or aggregates (excitation of 585 nm and emission of 590 nm) using the fluorescence microscope. Mitochondrial depolarization is distinguished by a decrease in the red/green fluorescence intensity ratio.

### Animals and experimental design

C57BL/6 female mice with similar body weight used in this study were purchased from Center for Experimental Animals, Guangdong Province [Guangzhou, China approval number SCXK (Yue) 2013-0002]. The mice were housed in a standard day and night cycle with free access to food and water. These animals were mainly used for three parts of the experiment consisting of pharmacodynamics, toxicology and biodistribution of drugs *in vivo*. All animal experiments were performed in accordance with the Guidelines for the Care and Use of Experimental Animals, the Guangdong Pharmaceutical University [SYXK (Yue) 2012-0125] and approved by the Guangdong Pharmaceutical University Animal Care and Use Committee, China. A total of 82 mice were randomly divided into six groups in pharmacodynamic study: normal group (n = 10, Normal), model group (n = 15, Control), mesoporous carbon nanospheres group (n = 15, MCNs), *Musca domestica* cecropin group (n = 15, MDC), MDC@MCNs group (n = 15, MDC@MCNs) and sulfasalazine group (n = 12, SASP). The experiment design was shown in **Figure 6A**. The entire experiment period continued for 11 days (day -1 to 9). The physiological state and body weight changes of mice were recorded in detail throughout the period to evaluate the disease activity index (DAI). The specific rule of DAI scoring associating with the severity of body weight loss, loose stool and gross bleeding was consistent with the description of previous study **80**. Finally (day 10), the mice were killed to obtain serum and tissues for the follow-up experiments in accordance with the relevant ethical requirements. UC model was induced by free drinking 2.5% DSS water solution for 8 days (day 0 to 7) and the mice with UC were orally given with 0.5% sodium carboxymethylcellulose (CMC-Na). The mice in normal group received drinking water and also obtained the treatment with 0.5% CMC-Na. The dosage and method of administration for the other groups were as follows: MDC powder was dissolved in normal saline and injected intraperitoneally at a dose of 2 mg/kg. Considering the involvements of drug loading and release rate as well as theoretical difference of bioavailability between intraperitoneal injection and oral administration, MCNs and MDC@MCNs were homogenized in 0.5% CMC-Na solution and given by means of intragastric administration at a dose of 25 mg/kg (equivalent to the same available dose of MDC). Similarly, 0.5% CMC-Na was used as solvent for sulfasalazine enteric coated tablets with the corresponding oral dose of 300 mg/kg. In the toxicology experiment for a period of two weeks, 24 mice were randomly assigned to four groups (n = 6): normal group, MCNs group (125 mg/kg), MDC group (10 mg/kg) and MDC@MCNs group (125 mg/kg). The administration method of each group was the same as that of pharmacology study, and the only difference was that the dose was 5 times of the previous one. In addition, 4 mice and 14 mice were used to assess intestinal adhesion of MCNs and monitor oral biodistribution of FITC-MDC and FITC-MDC@MCNs in alimentary canal, respectively.

### Hematoxylin-eosin (H&E) staining and transmission electron microscopy (TEM)

The entire colon (from the cecum to the anus) was removed, while the colon length was measured standing for the damage and inflammation indirectly. The distal colon was used for histological analysis. The heart, liver, spleen, lung and kidney of each group of mice used for toxicological assessment were isolated. The above small tissue segments were fixed in 4% paraformaldehyde and embedded with paraffin. 5-μm thickness sections were obtained from the paraffin blocks and adhered to the slides to be processed via hematoxylin-eosin (H&E) staining. The photomicrographs were acquired under light microscopy with a digital camera (Zeiss, Oberkochen, Germany). Histopathologic scores of colons were evaluated by three different observers using the criteria described previously 81. In addition, the changes of the ultrastructure of colon in each group were further observed by transmission electron microscopy (TEM). Briefly, a cubic millimeter of colonic tissue was fixed in 2.5% glutaraldehyde at 4°C overnight and then in aqueous 1% OsO_4_ for 2 h. Subsequently, the tissue was treated with 2% uranyl acetate for 1 h after dehydration in an ethanol series and embedded in epoxy resin. Sections finally were photographed using a JEM1400 transmission electron microscope (Jeol, Tokyo, Japan). The grading standard of colonic microstructure damage was implemented according to the previous study 8.

### Enzyme-linked immunosorbent assay (ELISA)

To demonstrate effect of drugs on inflammatory and immunological responses in UC mice, the cytokines and chemokines in serum and colon tissues such as Tumor Necrosis Factor-α (TNF-α), Interferon-γ (IFN-γ), Interleukin-6 (IL-6), Interleukin-1β (IL-1β), Interleukin-17 (IL-17), Interleukin-10 (IL-10) and Diamine Oxidase (DAO) were determined by using enzyme linked immunosorbent assay (ELISA). All detection steps and calculation methods were carried out according to the instructions of the ELISA kits (Andy Gene Biotechnology Co. Ltd, Beijing, China). The colonic samples were added to a pre-cooled saline in a ratio of 1:9 (0.1 g/0.9 mL). The tissues were cut broken by high-pressure sterilized surgical scissors and homogenized as fully as possible. The supernatants after centrifugation were prepared for total protein concentration determination according to the BCA assay kit (Beyotime, Shanghai, China) and then taken for the ELISA test. All values were expressed as picograms/milliliter (pg/mL).

### CAT, MPO, MDA, SOD, GSH-Px, AST, ALT, BUN and CRE detection

Colonic Catalase (CAT), Myeloperoxidase (MPO), Malondialdehyde (MDA), Superoxide Dismutase (SOD) and Glutathione Peroxidase (GSH-Px) levels were determined by using corresponding kit provided by Nanjing Jiancheng Bioengineering Institute (Nanjing, China). The preparation method of colon supernatants was the same as that of ELISA. Moreover, Glutamic Oxaloacetic Transaminase (AST), Glutamic Pyruvic Transaminase (ALT), Blood Urea Nitrogen (BUN) and Creatinine (CRE) concentrations in serum of mice in toxicology experiment were detected by detection kits, which were also purchased from Nanjing Jiancheng Bioengineering Institute. All experimental steps and data processing were carried out according to the instructions.

### Erythrocyte hemolysis evaluation

To evaluate erythrocyte hemolysis of MCNs under different concentration (the dosage of anti UC action was covered in this range), the blood of human (donated by healthy volunteers), rats and mice was collected by anticoagulant tube, centrifuged at 1200 g for 15 min and then washed with PBS for three times to obtain fresh red blood cells. MCNs were suspended in PBS to form suspensions with different concentrations (0.1, 0.2, 0.4, 0.8, 1, 2, 4 mg/mL). In addition, because pharmacological and toxicological experiments were actually carried out in C57BL/6 mice, we focused on the effects of each administration group (the actual dose was higher than that in the pharmacological experiment) on the erythrocytes of mice. The concentration of MDC, MCNs and MDC@MCNs suspended in PBS was 1 mg/mL, 3 mg/mL and 3 mg/mL respectively. The 150 μL above suspension was mixed with 150 μL prepared 2% red blood cells. The red cells treated with 1% Triton X-100 and PBS were used as the positive control group and negative control group respectively. The mixed suspensions were heated in 37 °C water bath for 1h, then centrifuged for 15 min, and finally photographed as a hemolysis observation record.

### Quantitative real time reverse transcription-polymerase chain reaction (qPCR)

Total RNA samples from the distal colons were purified and reversely transcribed into cDNA using Trizol reagent (Invitrogen Corporation, Carlsbad, USA) and PrimeScript^TM^ RT reagent Kit with gDNA Eraser (Takara Biotechnology Co. Ltd., Kusatsu, Japan), respectively. Quantitative Real-time PCR (qPCR) was conducted to detect the mRNA expression of individual genes using the SYBR Premix Ex Taq Kit (Takara Biotechnology Co. Ltd., Kusatsu, Japan) in the CFX Connect fluorescence quantitative PCR detection system (BIO-RAD, Hercules, USA). Data were analyzed according to the comparative threshold cycle (Cq) method and normalized to an endogenous reference, housekeeping gene β-actin. The primers used in this experiment were listed as follows:

- ZO-1 forward: 5′-CCAGCAACTTTCAGACCACC-3′, reverse: 5′-TTGTGTACGGCTTTGGTGTG-3′;
- Claudin-1 forward: 5′-TCGACTCCTTGCTGAATCTGA-3′, reverse: 5′-TCCACATCTTCTGCACCTCA-3′;
- Occludin forward: 5′-GCTTACAGGCAGAACTAGACG-3′, reverse 5′-TCTGCAGATCCCTTAACTTGC-3′;
- NF-κB p65 forward: 5′-TCTTCTTGCTGTGCGACAAG-3′, reverse: 5′-GCATGGAGACTCGAACAGGA-3′;
- COX-2 forward: 5′-AGGTCATTGGTGGAGAGGTG-3′, reverse: 5′-CCTGCTTGAGTATGTCGCAC-3′;
- INOS forward: 5′-ACAGGAACCTACCAGCTCAC-3′, reverse: 5′-CGACCTGATGTTGCCATTGT-3′;
- β-actin forward: 5′-AGAGGGAAATCGTGCGTGAC-3′, reverse: 5′-CAATAGTGATGACCTGGCCGT-3′.

### Western blotting

Distal colons were ground and lysed in RIPA lysis buffer (Beyotime, Shanghai, China) containing 1 mM PMSF (Beyotime, Shanghai, China) to extract proteins. The protein samples were quantified using BCA method and diluted to a certain concentration. Equal amounts of denaturated protein (20 μg/well) were loaded in each lane of a 5/12% or a 5/8% SDS-PAGE gel. The proteins were separated by SDS-PAGE and transferred onto PVDF membranes. The membranes were blocked with 5% (w/v) defatted milk for 1 h at room temperature and incubated with primary antibodies (Abcam, Cambridge, UK) such as NF-κB p65 (1:1000), COX-2 (1:1000), INOS (1:1000), ZO-1 (1:1500), Claudin-1 (1:1000), Occludin (1:1000) overnight at 4 °C. HRP-conjugated secondary antibody (1:1000) (Beyotime, Shanghai, China) was applied to the membranes before the signals were visualized via chemiluminescence using ECL luminescence reagent (Beijing 4A Biotech Co., Ltd, Beijing, China). Images were collected with Tanon-5200 (Shanghai Tanon Technology Co., Ltd, Shanghai, China) scanning the membranes under the best exposures. The densities of the bands were determined with Image J software and normalized to band intensity for GAPDH. Each experiment was repeated at least three times.

### 16s rDNA sequencing

Colon contents were collected strictly according to sample collection standards for 16s rDNA sequencing. High-throughput sequencing of the 16s V4 region revealed the the difference of species composition between groups and the relationship between flora and corresponding indexes in this study. All the sequencing procedures including quality inspection of samples, DNA extraction, sequencing by synthesis and general data analyses were performed by a commercial company (Novogene, Beijing, China).

### Intestinal adhesion of MCNs and oral biodistribution of FITC-MDC and FITC-MDC@MCNs in gastrointestinal tract

To verify the intestinal adhesion of MCNs, we assessed the accumulation difference of MCNs in colon of normal mice (blank group and MCNs group) and UC mice (blank group and MCNs group). In short, 20 mg MCNs was mixed in 0.5% CMC-Na solution of 2 mL and each mouse in MCNs group was then orally given about 500 μL of the drug. The each mouse in blank group was infused with 500 μL of 0.5% CMC-Na. After 4 hours, the entire colon of the mice was removed, cut open and rinsed repeatedly with normal saline. The black staining of the colon wall was observed and photographed. Meanwhile, to clarify oral biodistribution of FITC-MDC and FITC-MDC@MCNs in gastrointestinal tract (GIT) and further evaluate the colon tissue-accumulating delivery potential of MDC@MCNs, fourteen mice with normal condition were randomly assigned to FITC-MDC group and FITC-MDC@MCNs group and administrated with FITC-MDC (8.25 mg/kg) or FITC-MDC@MCNs (25 mg/kg) through oral administration, respectively. It was worth noting in the case of considering the drug loading rate that the initial dose of a given FITC-MDC (fluorescence intensity) remained consistent. Live *in vivo* fluorescent bioimaging was conducted on mice at the predetermined time points (2, 4, 6, 8, 10, 12 and 24 h). Meanwhile the mice were euthanized and the entire GIT was isolated for *ex vivo* imaging after live *in vivo* fluorescent bioimaging. Tanon 5200 Multi-Imaging System (Tanon, Shanghai, China) was used to measure FITC bioluminescence from FITC-MDC and FITC-MDC@MCNs in GIT of mice. The representative pictures were obtained and the related analysis was carried out with the supporting software. All the experiments above were repeated three times independently.

### Statistical analysis

All data were expressed as means ± SEMs of at least three independent assays. Statistical analyses were carried out using GraphPad Prism 7.0 (GraphPad Software Inc., La Jolla, USA). Differences between groups were analyzed by Student's t-test or one way ANOVA. *P* < 0.05 was considered statistically significant.

## Abbreviations

MDC: *Musca domestica* cecropin
MCNs: mesoporous carbon nanoparticles
UC: ulcerative colitis
BET: Brunauer Emmett Teller
XRD: X-ray diffraction
TEM: transmission electron microscope
PDI: polydispersity index
DSS: dextran sulphate sodium
MTT: 3-(4,5-Dimethylthiazol-2-yl)-2,5-diphenyltetrazolium bromide
HE: hematoxylin-eosin
ELISA: enzyme linked immunosorbent assay
qPCR: quantitative real-time PCR
ROS: reactive oxygen species
AST: glutamic oxaloacetic transaminase
ALT: glutamic pyruvic transaminase
BUN: blood urea nitrogen
CRE: creatinine
MMP: mitochondrial membrane potential
TNF: tumor necrosis factor
IFN: interferon
IL: interleukin
DAO: diamine oxidase
Th: T helper cell
Treg: T regulatory cell
MPO: myeloperoxidase
CAT: catalase
MDA: malondialdehyde
SOD: superoxide dismutase
GSH-Px: glutathione peroxidase
COX: cyclooxygenase
INOS: inducible nitric oxide synthase
FITC: fluorescein isothiocyanate
